# Study on the Polymer Morphology and Electro-Optical Performance of Acrylate/Epoxy Resin-Based Polymer-Stabilized Liquid Crystals Based on Stepwise Photopolymerization

**DOI:** 10.3390/polym16172446

**Published:** 2024-08-29

**Authors:** Yishuo Wu, Guangyang Shang, Cong Ma, Yingjie Shi, Zhexu Song, Peixiang Wang, Yanzi Gao, Qian Wang, Meina Yu, Jiumei Xiao, Cheng Zou

**Affiliations:** 1 Beijing Advanced Innovation Center for Materials Genome Engineering, Institute for Advanced Materials and Technology, University of Science and Technology Beijing, Beijing 100083, China; m202111387@xs.ustb.edu.cn (Y.W.); shangguangyang@126.com (G.S.); syj0625@163.com (Y.S.); songzhexu1108@163.com (Z.S.); gaoyanzi@ustb.edu.cn (Y.G.); b2286713@ustb.edu.cn (Q.W.); yumeina@ustb.edu.cn (M.Y.); 2 Strategic Business Unit of Chlor-Alkali, Sinochem Group, Beijing 100031, China; macong133@126.com; 3 Yantai Xianhua Technology Group Co., Ltd., Yantai 264006, China; wangpx100@163.com; 4 School of Mathematics and Physics, University of Science and Technology Beijing, Beijing 100083, China

**Keywords:** PSLCs, acrylate, epoxy resin, electro-optical properties, polymer morphology

## Abstract

Stepwise photopolymerization is a miraculous strategy modulating the polymer skeleton and electro-optical properties of light modulators based on liquid crystal/polymer composites. However, owing to the indistinct polymerization mechanism and curing condition discrepancy, the required polymer structures and electro-optical properties are hard to be controlled precisely. Herein, a novel polymer-stabilized liquid crystal film based on acrylate/epoxy resin is proposed, fabricated and the relationships between preparation process, polymer content, polymer morphology and electro-optical properties are studied. The in-situ photopolymerization of acrylate/epoxy resin liquid crystalline polymer is fulfilled using cation photo-initiator UV 6976. The distinct photopolymerization speed between acrylate and epoxy resin benefits the polymer morphology control, and with accurate containment of the polymerization process and polymer composition, the superior electro-optical properties at a higher polymer content are acquired. The polymer morphology and electro-optical properties are influenced by the polymer content and mass ratio between acrylate and epoxy resin. The best electro-optical properties among samples are attained by controlling the mass ratio between acrylate and epoxy resin to 1:1, integrating higher densities of scattering centers and lower anchoring effect. With higher polymer content, the strategy of increasing the mass ratio of E6M benefits the improvement of E-O properties for alleviating polymer density. This work provides insights to stepwise polymerization of liquid crystalline monomers and offers a fancy strategy for the preparation of novel liquid crystal dimming films.

## 1. Introduction

The greenhouse effect has always been a burning threat to human’s subsistence, hence much attention and efforts aimed at yielding and saving energy massively have been proposed [1,2]. According to statistics, in developed countries, the indoor energy consumption and carbon emissions, dividing mainly into construction and transportation, occupied over 60% of the total [3,4]. Smart window, which has always been an energy-saving option nowadays in an industrial society, by sensing the outdoor environment thereby controls light transmittance hence energy importation instantly to suit human’s indoor environment demand [5,6]. Among the products made of various material systems, including electrochromics [7], photochromics [8], thermochromics [9], hydrogels [10] and liquid crystal (LC)/polymer composite dimming films [11], the LC device took advantage of high cost-performance efficiency, accessibility to large-scale flexible production and stability along with wide adaptability [12].

Usually, there were normal-mode dimming film and reverse-mode dimming film among the LC/polymer composite films, where the normal-mode meant being opaque at off-state and clear at on-state, while the reverse-mode meant clear at off-state and opaque at on-state [13]. The normal-mode dimming film, mainly made of polymer-dispersed liquid crystals named after the composite structure of dispersing the liquid crystal microdroplets into polymer matrix, has penetrated in practical application since it has high mechanical properties from high polymer content [14,15]. However, its drawback of a sideview haze from the mismatching of refractive index between polymer and LC as well as high driving voltages hampered its broader prospect. The most unacceptable issue is the deadly security threat of a sudden outage, when the vision is lost immediately with no chance to recover spontaneously [16]. The reverse-mode dimming film was mainly based on polymer stabilized liquid crystal (PSLC), which was named after the anchoring stabilization from aligned liquid crystalline polymer on liquid crystal. The PSLC film possessed the energy-saving feature, low driving voltages along with high contrast ratio as well as free-off sideview haze, and most importantly avoided the security threat at sudden outage [17,18], which showed broader application potential and prospect in energy-saving smart windows. However, its contradiction between high mechanical performance and E-O properties hampered its accessibility toward application [19]. On the one hand, the polymer content was low due to the sharply increasing anchoring force and driving voltages with the increase in polymer content [20]. On the other hand, the fragile polymer texture and weak adhesion at the substrate surface impaired the loading mode [21]. As a consequence, the PSLC films had poor mechanical properties and processibility.

To solve the critical issue, Liang et al. proposed a polymer-dispersed and stabilized liquid crystal with both the structure and performance of PDLC and PSLC, integrating gratifying E-O properties and mechanical properties [22]. However, the structure essence of PDLC brought the sideview haze, and the high frequency electric field caused a serious heating effect. Zhang et al. demonstrated the alleviated texture tension by decreasing polymerization speed to foster sufficient chain relaxation via reversible addition–fragmentation chain transfer (RAFT) polymerization. However, the polymer content was still limited [23]. Li et al. refined the polymer wall-stabilized liquid crystal, integrating larger polymer content and satisfactory E-O properties by modulating the structure of the periodic aligned polymer wall [20]. Chen et al. studied the relationship between the fabrication condition and E-O properties of epoxy resin-based PSLC, where the device inherited the strong mechanical properties of epoxy resin rubber conferring high cyclic stability [24]. Yoon et al. fabricated the liquid crystal light modulator with periodic homeotropic aligned polymer chamber, which benefited the load mechanism and mechanical properties [25]. Nevertheless, a PSLC with good E-O properties and high mechanical properties is an urgent need, which has been a difficult task to achieve both.

Stepwise polymerization has always been a resounding solution for the design of multiphase polymer microstructure and properties. Guo et al. firstly proposed a PD&SLC coexistence system by stepwise photopolymerization using non-liquid crystalline monomers and liquid crystalline monomers, forming PDLC skeleton at first and then PSLC in LC microdroplets [26]. As the monomers were all acrylates, the curing condition and polymerization speed difference is indistinct, and the required polymer structures and electro-optical properties were hard to be controlled precisely. Previous studies showed acrylate-based PSLC possessed superior E-O properties, while the polymer was limited to a very low level and fragile. Epoxy resin possessed superior mechanical properties. Most importantly, the polymerization conditions could be quite different for these two kinds of monomers.

In this study, a PSLC based on acrylate/epoxy resin polymer harnessing acrylate and epoxy resin liquid crystalline curable monomer was fabricated by free-radical in-situ photopolymerization and cation in-situ photopolymerization simultaneously in a uniform system. The photopolymerization with cation photoinitiator UV 6976 at high UV intensity yielded free-radical particle and cation simultaneously and the stepwise polymerization of two polymer was fulfilled through the discrepancy between suitable polymerization mechanism and speed. The effects of curing conditions, initiator and monomer compositions, monomer content on the polymer morphologies and electro-optical properties of PSLC films have been systematically studied. The mass ratio of C6M and E6M played a vital role in the polymer morphology and E-O properties. Only when the mass ratio of C6M and E6M approached, the superior E-O properties were more likely to be achieved, resulting from moderate fiber density and scattering centers. The PSLC with acrylate/epoxy resin polymer showed a new type of polymer morphology and good E-O properties at higher polymer content, which supplied precious reference toward theoretical research and practical application of PSLC-based reverse-mode dimming film.

## 2. Materials and Methods

### 2.1. Materials

**Figure 1:**
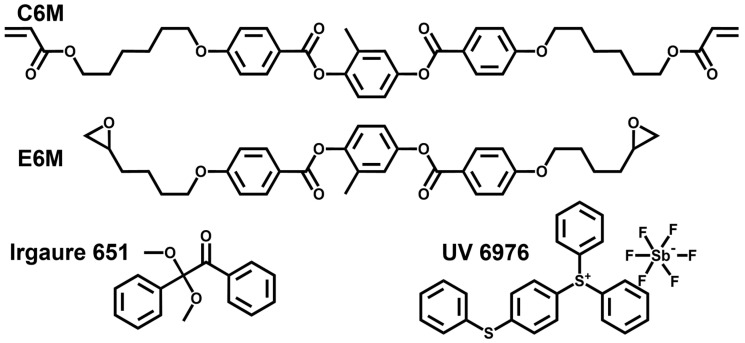
The molecular structures of materials used in this study.

An overview of the molecular structures of materials used in this study is given in Figure 1. The negative nematic liquid crystals (NLCs) used in the experiments are GXV-7822-180 (T_NI_ = 380.15 K, Δε = −4.4, Yantai Xianhua Chemical Technology Co., Ltd., Yantai, China). The liquid crystal monomer C6M was purchased from Jiangsu Hecheng Advanced Materials Co., Ltd., Nanjing, China. E6M was purchased from Beijing Gerui Kechuang Technology Co., Ltd., Beijing, China. The photo initiator Irgacure 651 was bought from TCI Co., Ltd., Shanghai, China. The cationic photo-initiator UV 6976 was purchased from Shanghai Macklin Biochemical Technology Co., Ltd., Shanghai, China.

### 2.2. Sample Preparation and Measurement

First of all, all materials used, containing LC, liquid crystalline monomer, free-radical photoinitiator and cation photoinitiator were stirred fiercely according to the compositions in Table 1 to derive a homogeneous mixture. Then the mixture was injected into the homeotropic alignment LC cell with a cell thickness of 8 μm through capillary. The LC cell was then exposed to the UV light at the certain intensity and time.

The E-O properties were tested by a liquid crystal device parameter tester LCT-P1000 (Changchun Liancheng Instrument Co., Ltd., Changchun, China) at room temperature. The incident light source was a halogen laser (λ = 560 nm), the applied electric field was a square wave (50 Hz) and the signal after passing through the sample was collected by a photodiode detector with the collection angle controlled at ±2°. The E-O properties of PSLC included several parameters. The T-V curve was derived observing the light transmittance instantly at the exact voltage applying a gradient uplifting voltage with a certain time period. Relevant E-O parameters could be calculated from the T-V curve: threshold voltage (V_th_), which meant the voltage decreased by 10% transmittance of the total variation; saturation voltage (V_sat_), which meant the voltage decreased by 90% transmittance of the total variation; contrast ratio (CR), which meant the ratio between the highest and lowest transmittance. The response times were monitored with a digital storage oscilloscope. On-state response time (T_on_) meant the time spent on decreasing the transmittance 90% of the total variation after applying a voltage; off-state response time (T_off_) meant the time spent on the transmittance increasing 90% of the total variation after removing the voltage. The transmittance of air was normalized at 100%. The polymer microstructures were observed by using scanning electron microscopy (SEM, HITACHI S-4800). Before observation, the prepared samples were split into small blocks of about 1–2 cm squares and immersed in cyclohexane for about 7 days. Cyclohexane was required to be replaced regularly to remove the liquid crystal molecules more thoroughly. Then, the samples were dried in a vacuum oven at 60 °C for 24 h. Finally, after the gold spray treatment on its surface, the samples were used for the SEM observation.

## 3. Results and Discussion

### 3.1. The Impact of the Photopolymerization Condition on the Polymer Morphology and E-O Properties

**Figure 2:**
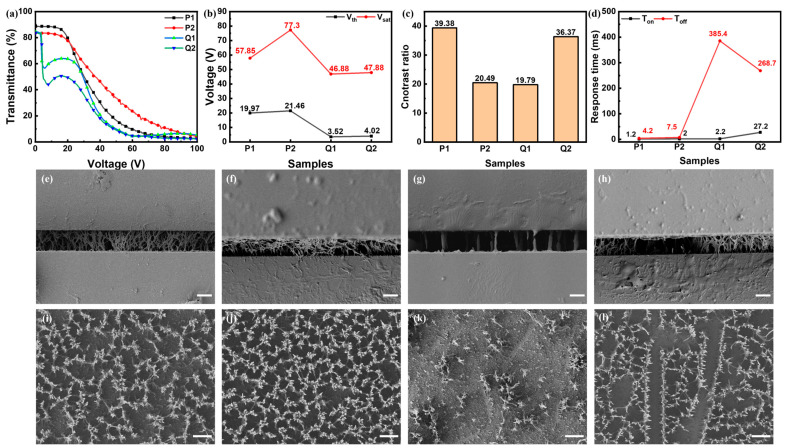
(**a**–**d**) E-O properties of samples in group P and Q: (**a**) T-V curves, (**b**) driving voltages, (**c**) contrast ratio, (**d**) response times; (**e**–**h**) side-view SEM photos of (**e**) P1, (**f**) P2, (**g**) Q1, (**h**) Q2; (**i**–**l**) top-view SEM photos of (**i**) P1, (**j**) P2, (**k**) Q1, (**l**) Q2. All the scale bars are 5 μm.

PSLC based on acrylate/epoxy resin polymer was proposed in this research, where the priority was the polymerization process. The polymerization of acrylate liquid crystalline monomer was based on free-radical in-situ polymerization demanding free-radical particles, whereby the epoxy resin liquid crystalline polymer required cation as catalysts. The cation photoinitiator UV 6976 yielded free-radical particles and cations simultaneously, and the schematic representation of the crosslinking reaction of E6M and C6M is shown in Figure S1. A polymer content of 4 wt% C6M + 4 wt% E6M was selected, and four polymerization processes were researched. The group P contained Irgaure 651, while group Q contained none. P1 and Q1 were irradiated by UV light at 25 mW/cm^2^ for 10 min at first, and then 75 mW/cm^2^ for 2 h, while P2 and Q2 75 mW/cm^2^ for 2 h. Figure 2a–d exhibits E-O properties of samples in group P and Q. First of all, the driving voltages in group P were much higher than those in group Q, and driving voltages of P1 were much smaller than P2. In addition, the contrast ratio of P1 and Q2 was relatively higher than P2 and Q1. Lastly, the response times of group P were several milliseconds, while in group Q the response times t_on_ were several milliseconds and t_off_ was hundreds of milliseconds. The E-O properties resulted from polymer morphologies. The top-view and side-view SEM images of group P and Q were shown in Figure 2e–l. First of all, the polymer densities of samples in group P were much higher than those of group Q, hence yielding a much higher anchoring force, which is why the driving voltages of group P were much higher. That probably resulted from a much higher free-radical generation rate with the addition of Irg 651. No matter the UV intensity of 25 or 75 mW/cm^2^, the decomposition speed of Irg 651, generation speed of free-radical particles as well as the polymerization speed of acrylate were swift enough. In addition, theoretically, the fulfillment of cation polymerization of epoxy resin went much slower than acrylate, as was verified that the polymer morphology was mainstreamed by acrylate polymer. The comparatively sparser polymer densities of samples in group Q were speculated to have a comparatively lower free-radical particle generation rate only utilizing UV 6976, where the sparser polymer density of Q1 was from the lower free-radical generation rate at a lower UV intensity. The higher contrast ratios of P1 and Q2 were probably from the moderate density of polymer, where the dense scattering centers formed along dense liquid crystal domains under moderate anchoring force. The lower contrast ratio of Q1 compared to Q2 was due to deficient scattering densities originated form the much sparser polymer networks. The comparatively low T_off_ in group P was probably from that higher polymer density which offered higher anchoring force to liquid crystal molecules to rotate back to the original state. The FT-IR spectra of Q2 at different polymerization time is given in Figure S2. The peaks referring C=C double bond with a wave number 1636 cm^−1^ and epoxy with a wave number of 906 cm^−1^ disappeared completely after 2 h, which indicated the completed polymerization of C6M and E6M.

### 3.2. The Impact of Mass Ratios between C6M and E6M on Polymer Morphologies and E-O Properties

**Figure 3:**
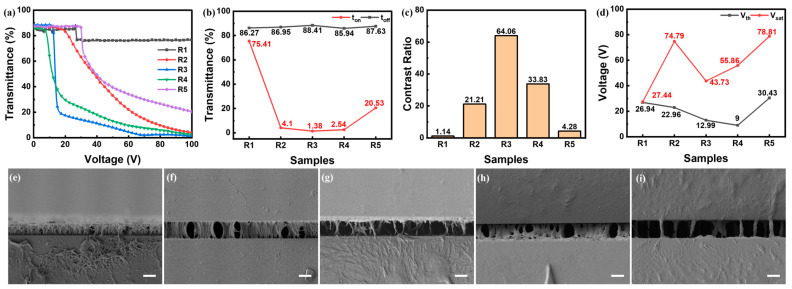
(**a**–**d**) E-O properties of samples in group R: (**a**) T-V curves, (**b**) off-state and on-state transmittance, (**c**) contrast ratio, (**d**) driving voltages; (**e**–**i**) side-view SEM photos of Sample (**e**) R1, (**f**) R2, (**g**) R3, (**h**) R4 and (**i**) R5, where all scale bars are 5 μm.

The impact of the mass ratios between E6M and C6M on the polymer morphology and E-O properties were studied, and two groups of samples with different polymer contents of 10 wt% and 12 wt% were fabricated and characterized. In each group, the mass ratio between E6M and C6M varied. The E-O properties of samples in group R were shown in Figure 3a–d. With a high mass ratio of C6M or E6M, R1 and R5 had poor E-O properties, manifested by high transmittance at on-state and a lower contrast ratio. As the mass ratio between C6M and E6M approached 1:1, the E-O properties of R2 and R4 improved compared to R1 and R5, where the E-O properties of R2 were slightly inferior to R4, in aspects of driving voltages and contrast ratio. Among them, R3 with the mass ratio of 1:1 fetched the best E-O properties.

This E-O properties variation explained the relationship between polymer density and scattering center decided by polymer morphology with the variation in polymer contents. As seen in the SEM photo of R1 in Figure 3e, the higher mass ratio of C6M yielded denser polymer, offering overdose anchoring force largely hindering LC molecules rotating to form the scattering state. As the mass ratio of C6M decreased and E6M increased, the polymer density and thereby anchoring force declined as seen in Figure 3f, and the E-O properties of R2 benefited a little compared to R1. With the highest mass ratio of E6M, the polymer had separately sparse fibers as shown in Figure 3i. Too sparse scattering centers led to higher transmittance even with a voltage of 100 V. As for sample R4, the polymer density grew denser with increased acrylate polymer, where it enriched the scattering density and benefited the E-O properties. The E-O properties of R4 were superior to R2, since the polymer density was sparser, hence holding less anchoring force as well as more liquid crystal domains for scattering centers. Among them, the E-O properties of R3, with the mass ratio between C6M and E6M of 1:1, exhibited the best among this group. As seen in Figure 3g, the liquid crystal domain density was higher, which was beneficial for better E-O properties.

**Figure 4:**
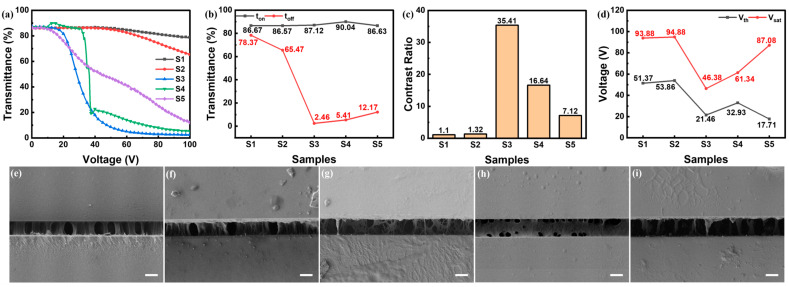
(**a**–**d**) E-O properties of samples in group S: (**a**) T-V curves, (**b**) off-state and on-state transmittance, (**c**) contrast ratio, (**d**) driving voltages; (**e**–**i**) side-view SEM photos of Sample (**e**) S1, (**f**) S2, (**g**) S3, (**h**) S4 and (**i**) S5, where all scale bars are 5 μm.

When the polymer content rose up to 12 wt%, the regulation of E-O properties of samples in group S with the variation in the mass ratio between C6M and E6M somewhat imitated the group R, as shown in Figure 4. The E-O properties of S1 imitated R1, the transmittance was hard to decrease, since the over dense polymer was mainstreamed by the content of acrylate. As the mass ratio of E6M increased, the E-O properties of S2 grew a little better than S1 as well as inferior to R2, where the polymer density was observed to be dense as shown in Figure 4f. That verified again that the polymer morphology and E-O properties were sculptured by monomer composition. At another side, the contrast ratio of S5 with a high mass ratio of E6M was similarly low among this group as in group R. The polymer morphology was seen with similar sparsely separated fibers as R5, while the slightly higher scattering center created a slightly higher contrast ratio. The E-O properties of S4 were similarly superior to S5 and S2 as R4 to R5 and R2 in group R, as denser scattering centers benefited that. The E-O properties of S4 were slightly inferior to R4 by denser polymer and sparser scattering centers. That polymer morphology deduced epoxy resin to intuitively form at the wrinkle at fulfilled acrylate, hence more E6M occupied nearly all gaps of acrylate fiber. Similarly, the E-O properties of S3, with the mass ratio between E6M and C6M 1:1, acquired the best E-O properties in this group, seen by moderate polymer density and high scattering centers for intense scattering. Compared to R3, the E-O properties descended, which meant that more polymer content enlarged the anchoring force.

The variation in polymer morphologies along the mass ratio between E6M and C6M manifested the sculpture process and therein polymerization process. The polymer density increased along the increasing mass ratio of C6M, which indicated the acrylate polymer polymerization was fulfilled far ahead of epoxy resin and sculptured the skeleton of the polymer. At higher content of E6M, with the increasing mass ratio of C6M, the polymer morphologies turned higher connected fibers from separated fibers. Epoxy resin took conspicuous effect as a morphology modulator. For instance, S4 exhibited a distinct polymer morphology compared to R4 with the same C6M content of 4 wt%.

### 3.3. The Impact of Polymer Content on the Polymer Morphology and E-O Properties

The pursuit of increasing the polymer content has always been a predominant topic of PSLC for enhancing mechanical properties. It has been proven that the mass ratio of 1:1 fetched the best E-O properties among samples with same polymer content, and a higher mass ratio of C6M largely inflated the polymer morphology thereby larger anchoring force, producing poor E-O properties. However, in circumstances with lower mass content and ratio of C6M, the impact of acrylate polymer content as a skeleton on the polymer morphology and E-O properties has not been clarified, where it seemed that E6M incredibly modulated the polymer morphology. The introduction of C6M into E6M somewhat benefited the E-O properties, manifested by R4 and S4 to R5. Therefore, two groups of samples aiming at two directions toward the increase in polymer content were fabricated. The samples in group U increased the polymer content to 13 wt% and 15 wt% with the mass ratio between C6M and E6M of 1:1. The samples in group T set the E6M content of 10 wt% and C6M contents of 3 wt% and 5 wt%, matching the same polymer content in group U.

**Figure 5:**
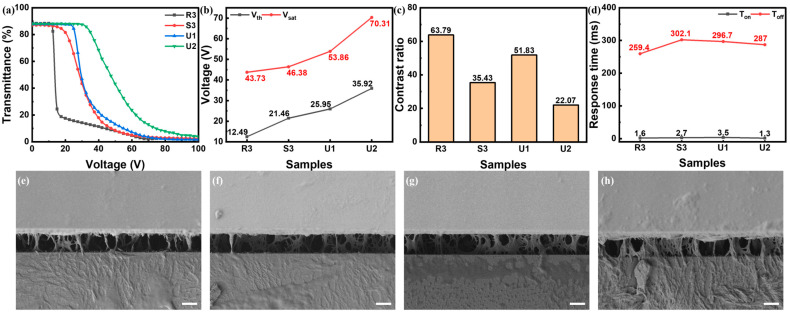
(**a**–**d**) E-O properties of samples with different polymer content: (**a**) T-V curves, (**b**) driving voltages, (**c**) contrast ratio, (**d**) response time; (**e**–**h**) side-view SEM photos of (**e**) R3, (**f**) S3, (**g**) U1, (**h**) U2, where all scale bars are 5 μm.

With the same mass ratio between E6M and C6M of 1:1, as the polymer content rose, the E-O properties deteriorated. As shown in Figure 5, the driving voltages increased with the increase in total polymer content. When the polymer content rose to 15 wt%, the V_sat_ increased to around 70 V while the contrast ratio dropped around 20. The SEM photos in Figure 5e–h indicated that the polymer networks showed a similar porous structure with denser polymer networks alongside the increase in the polymer content. This indicated that the denser polymer networks imposed a higher anchoring force on liquid crystal molecules thus leading to higher driving voltages.

**Figure 6:**
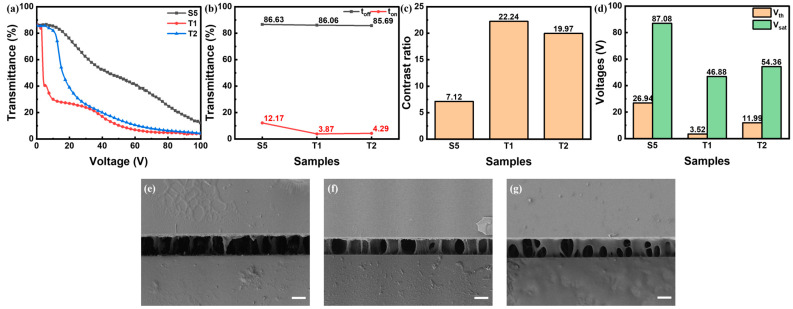
(**a**–**d**) E-O properties of S5, T1, T2: (**a**) T-V curves, (**b**) off-state and on-state transmittance, (**c**) contrast ratio, (**d**) driving voltages; (**e**–**g**) side-view SEM photos of sample (**e**) S5, (**f**) T1 and (**g**) T2, where all scale bars are 5 μm.

The driving voltages of U2 were too high for practical use. So, the group T samples with lower content of C6M were prepared and characterized. As shown in Figure 6, when the total content of monomer was 13 wt%, the threshold voltage of T1 was lower than U1, while the saturated voltage increased and contrast ratio was dragged down. This resulted from sparser densities of polymer as well as scattering centers as shown in Figure 5g and Figure 6f. The E-O properties of T2 were better than U2, with lower driving voltages and similar contrast ratio, which resulted from more appropriate densities of polymer and scattering centers as shown in Figure 6g. It verified more than one strategy for enhancing the E-O properties at higher polymer content. By introducing more E6M, the polymer network could be modulated to a more sparser feature thus reducing the anchoring force of polymer networks and reducing the driving voltages of PSLC with a high polymer content.

## 4. Conclusions

In conclusion, the acrylate/epoxy resin-based polymer stabilized liquid crystal was first proposed and realized by the stepwise photopolymerization method. The polymerization mechanism, process and suitable condition were studied at first, concluding that the curing condition of using UV 6976 and the UV intensity of 75 mW/cm^2^ for 2 h was the best polymerization condition. The relationships between the polymer composition and content, polymer morphology and E-O properties were clarified. The polymer morphology was directly modulated by the mass ratio of acrylate and epoxy resin regardless of the total polymer content. With the higher mass ratio of C6M, the polymer grew denser, while with higher mass ratio of E6M, the polymer grew sparser. The mass ratio between E6M and C6M of 1:1 generated the best E-O properties among samples with same polymer content by generating moderate densities of polymer and scattering centers. Aimed at increasing polymer content with conserved E-O properties, the strategy of increasing the mass ratio of E6M effectuated at higher polymer content for reducing the polymer density and driving voltages. This work provides new insights to polymer network modulation of PSLC films and develops a fancy strategy for the preparation of novel liquid crystal dimming films.

## Figures and Tables

**Table 1:** All compositions and UV processes used.

	Content (wt%)					UV Process
Samples	LC	E6M	C6M	Irg 651	UV 6976	
P1	91.6	4	4	0.2	0.2	25 mW/cm^2^, 10 min; 75 mW/cm^2^, 2 h
P2	91.6	4	4	0.2	0.2	75 mW/cm^2^, 2 h
Q1	91.6	4	4	0	0.2	25 mW/cm2, 10 min; 75 mW/cm^2^, 2 h
Q2	91.6	4	4	0	0.2	75 mW/cm^2^, 2 h
R1	89.8	2	8	0	0.2	75 mW/cm^2^, 2 h
R2	89.8	4	6	0	0.2	75 mW/cm^2^, 2 h
R3	89.8	5	5	0	0.2	75 mW/cm^2^, 2 h
R4	89.8	6	4	0	0.2	75 mW/cm^2^, 2 h
R5	89.8	8	2	0	0.2	75 mW/cm^2^, 2 h
S1	87.8	2	10	0	0.2	75 mW/cm^2^, 2 h
S2	87.8	4	8	0	0.2	75 mW/cm^2^, 2 h
S3	87.8	6	6	0	0.2	75 mW/cm^2^, 2 h
S4	87.8	8	4	0	0.2	75 mW/cm^2^, 2 h
S5	87.8	10	2	0	0.2	75 mW/cm^2^, 2 h
T1	86.8	10	3	0	0.2	75 mW/cm^2^, 2 h
T2	84.8	10	5	0	0.2	75 mW/cm^2^, 2 h
U1	86.8	6.5	6.5	0	0.2	75 mW/cm^2^, 2 h
U2	84.8	7.5	7.5	0	0.2	75 mW/cm^2^, 2 h

## Data Availability

The original contributions presented in the study are included in the article/Supplementary Materials, further inquiries can be directed to the corresponding authors.

## Supplementary Materials

The following supporting information can be downloaded at: https://www.mdpi.com/article/10.3390/polym16172446/s1, Figure S1. The schematic representation of the crosslinking reaction of E6M and C6M. Figure S2. The FT-IR spectra of Q2 at different reaction time.
