# Supplementary material for: Study on the Polymer Morphology and Electro-Optical Performance of Acrylate/Epoxy Resin-Based Polymer-Stabilized Liquid Crystals Based on Stepwise Photopolymerization
Source: Polymers (Basel). 2024 Aug 29;16(17):2446. doi: 10.3390/polym16172446 (PMC11398129; doi:10.3390/polym16172446)
Supplement: Supplementary file 1 [file polymers-16-02446-s001.zip › polymers-3167868-supplementary.pdf]

Supporting Information

Study on the Polymer Morphology and Electro-Optical Performance of Acrylate/Epoxy Resin-Based Polymer-Stabilized Liquid Crystals Based on Stepwise Photopolymerization

Yishuo Wu [1], Guangyang Shang [1], Cong Ma [2], Yingjie Shi [1], Zhexu Song [1], Peixiang Wang [3], Yanzi Gao [1], Qian Wang [1], Meina Yu [1], Jiumei Xiao [4,*] and Cheng Zou [1,*]

[1] Beijing Advanced Innovation Center for Materials Genome Engineering, Institute for Advanced Materials and Technology, University of Science and Technology Beijing, Beijing 100083, China; m202111387@xs.ustb.edu.cn (Y.W.); shangguangyang@126.com (G.S.); syj0625@163.com (Y.S.); songzhexu1108@163.com (Z.S.); gaoyanzi@ustb.edu.cn (Y.G.); b2286713@ustb.edu.cn (Q.W.); yumeina@ustb.edu.cn (M.Y.)

[2] Strategic Business Unit of Chlor-Alkali, Sinochem Group, Beijing 100031, China; macong133@126.com

[3] Yantai Xianhua Technology Group Co., Ltd., Yantai 264006, China; wangpx100@163.com

[4] School of Mathematics and Physics, University of Science and Technology Beijing, Beijing 100083, China

* Correspondence: jiujiu@sas.ustb.edu.cn (J.X.); zoucheng@ustb.edu.cn (C.Z.)

1. Schematic representation of the crosslinking reactions

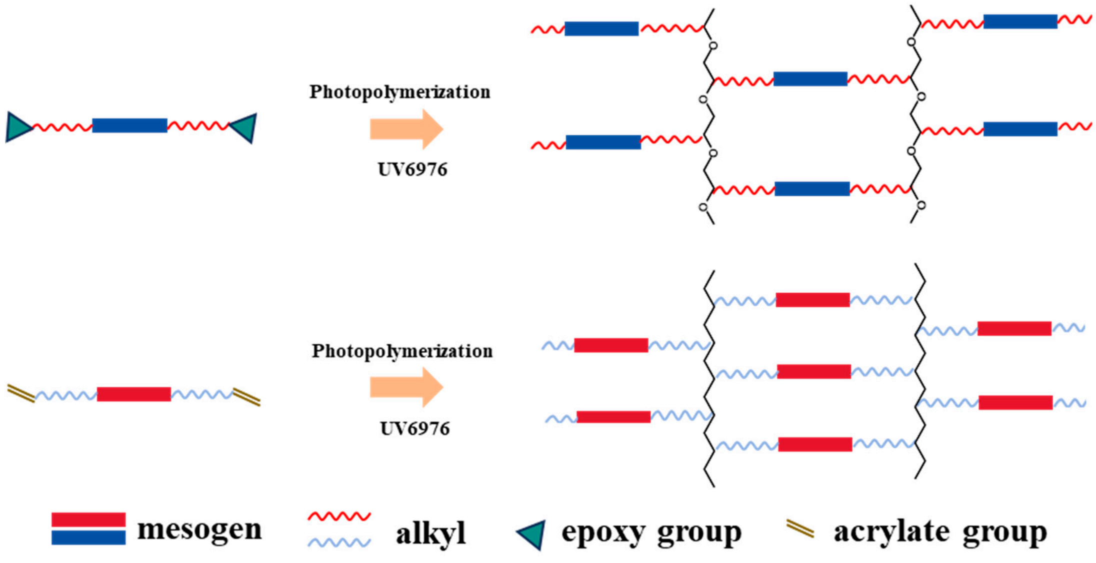

Figure S1. The schematic representation of the crosslinking reaction of E6M and C6M.

2. The FT-IR spectra of sample Q2 at different reaction time

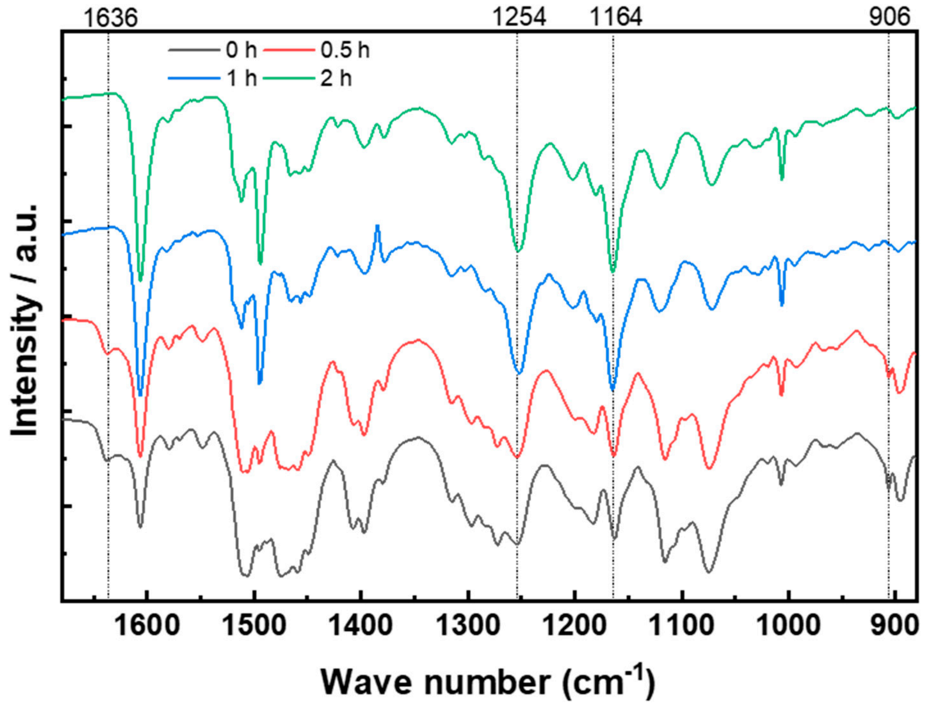

Figure S2. The FT-IR spectra of Q2 at different reaction time.
